# Aerobic exercise training rescues protein quality control disruption on white skeletal muscle induced by chronic kidney disease in rats

**DOI:** 10.1111/jcmm.13374

**Published:** 2017-12-19

**Authors:** Wilson Max Almeida Monteiro De Moraes, Pamella Ramona Moraes de Souza, Nathalie Alves da Paixão, Luís Gustavo Oliveira de Sousa, Daniel Araki Ribeiro, Andrea G. Marshall, Jonato Prestes, Maria Claudia Irigoyen, Patricia Chakur Brum, Alessandra Medeiros

**Affiliations:** ^1^ Biosciences Department Federal University of Sao Paulo Santos Brazil; ^2^ Post‐Graduation Program on Physical Education Catholic University of Brasilia (UCB) Brasilia Federal District Brazil; ^3^ Department of Post‐graduation in Medicine Nove de Julho University (UNINOVE) Sao Paulo Brazil; ^4^ School of Physical Education and Sport University of Sao Paulo Sao Paulo Brazil; ^5^ Department of Physiology and Biophysics Institute of Biomedical Sciences University of Sao Paulo Sao Paulo Brazil; ^6^ Department of Neurobiology Physiology and Behavior University of California Davis Davis CA USA; ^7^ Hypertension Unit Heart Institute University of Sao Paulo Medical School Sao Paulo Brazil

**Keywords:** chronic kidney disease, aerobic exercise training, skeletal muscle, protein quality control, unfolded protein response

## Abstract

We tested whether aerobic exercise training (AET) would modulate the skeletal muscle protein quality control (PQC) in a model of chronic kidney disease (CKD) in rats. Adult Wistar rats were evaluated in four groups: control (CS) or trained (CE), and 5/6 nephrectomy sedentary (5/6NxS) or trained (5/6NxE). Exercised rats were submitted to treadmill exercise (60 min., five times/wk for 2 months). We evaluated motor performance (tolerance to exercise on the treadmill and rotarod), cross‐sectional area (CSA), gene and protein levels related to the unfolded protein response (UPR), protein synthesis/survive and apoptosis signalling, accumulated misfolded proteins, chymotrypsin‐like proteasome activity (UPS activity), redox balance and heat‐shock protein (HSP) levels in the tibialis anterior. 5/6NxS presented a trend towards to atrophy, with a reduction in motor performance, down‐regulation of protein synthesis and up‐regulation of apoptosis signalling; increases in UPS activity, misfolded proteins, GRP78, derlin, HSP27 and HSP70 protein levels, ATF4 and GRP78 genes; and increase in oxidative damage compared to CS group. In 5/6NxE, we observed a restoration in exercise tolerance, accumulated misfolded proteins, UPS activity, protein synthesis/apoptosis signalling, derlin, HSPs protein levels as well as increase in ATF4, GRP78 genes and ATF6α protein levels accompanied by a decrease in oxidative damage and increased catalase and glutathione peroxidase activities. The results suggest a disruption of PQC in white muscle fibres of CKD rats previous to the atrophy. AET can rescue this disruption for the UPR, prevent accumulated misfolded proteins and reduce oxidative damage, HSPs protein levels and exercise tolerance.

## Introduction

CKD represents a major health burden for many countries, in part due to its aetiology that is based in highly prevalent diseases such as diabetes and hypertension. Otherwise, CKD is predominately asymptomatic, leading to late diagnosis and few options to improve prognosis 1.CKD is associated with lowered exercise capacity, which is a determinant for quality of life, negatively affecting the daily routine of patients 2. Indeed, lowered exercise capacity is an independent predictor of mortality in CKD 3. Therefore, its importance is in both quality of life and survival of these patients.

The determinants of lowered exercise capacity in CKD are multifactorial and include cardiac function, haemoglobin concentration and, mainly, skeletal muscle function 4. During the course of CKD, several factors can affect muscle function integrity such as metabolic acidosis, uraemic toxins, hormonal unbalance, nutritional status, muscle atrophy, inflammation and oxidative stress 4, 5, 6. Another condition that affects the homeostasis of skeletal muscle and impacts exercise capacity is reticulum stress 7, 8, 9. The endoplasmic reticulum (ER) is the major site in the cell for cellular Ca^2+^ storage, as well as lipid and protein synthesis, folding, assembly and trafficking. Certain stressful conditions, such as increased synthesis of secretory proteins, oxidative stress and inflammation, disrupt ER homeostasis and lead to the accumulation of misfolded proteins in the ER lumen. To cope with this stress, cells induce the UPR, which either repairs or degrades cytotoxic, damaged proteins using molecular chaperones and proteolytic systems, while attenuating protein translation 10.

The UPR is activated through three main transducers: ATF6 (activating transcription factor 6), IRE1α (inositol‐requiring enzyme 1 alpha) and PERK (protein kinase R‐like ER protein kinase). These proteins are normally bound by the chaperone binding immunoglobulin protein/glucose‐regulated protein 78 (BiP/GRP78) but are released upon UPR activation. The release of GRP78 results in activation of the kinases PERK and IRE1α. Once activated, PERK phosphorylates eukaryotic initiation factor 2 alpha (eIF2α), resulting in general protein translation attenuation and enhanced selective translation of the activating transcription factor 4 (ATF4). In turn, IRE1α causes splicing of X‐box binding protein 1 (XBP1) messenger RNA (mRNA). Translation of spliced XBP1 mRNA produces a transcription factor that up‐regulates specific target genes. ATF6 translocates to the Golgi complex where it is cleaved by proteases, releasing the cytoplasmic domain of ATF6 which activates transcription factors that regulate many processes related to protein folding and degradation 11.

When the UPR fails or is insufficient, the result is cell death, usually in the form of apoptosis. The transcription factor CCAAT/enhancer binding protein homologous protein (CHOP) is the main regulator of ER stress‐induced apoptosis and can be up‐regulated by the three aforementioned pathways 10. Therefore, therapeutic strategies that counteract UPR disruption are of great interest as they can offer more protection against stressor agents and contribute to tissue integrity.

One of the therapeutic strategies that has received much attention in CKD is AET, as it reduces uraemia symptoms and inflammatory and oxidative stress markers. This results in a more oxidative phenotype and improved skeletal muscle function, exercise capacity and quality of life in patients 12, 13. Of interest, different protocols of exercise training have been able to prevent the deleterious effects of UPR‐induced proteotoxicity and re‐established the PQC in different animal tissues 14, 15, 16, 17. Exercise‐induced UPR activation has been observed in skeletal muscles of both animals 9, 18 and humans 8. It is postulated that an UPR integrates the mechanisms of adaptation to exercise 9 and could prevent ER homeostasis disruption 16, as a single moderate‐intensity exercise session was able to activate the UPR in skeletal muscle, whereas that activation was attenuated after several exercise sessions 9, 18.

Despite its therapeutic potential and ability to modulate the UPR, the effects of AET on skeletal muscle disorders induced by CKD remain unknown. Thus, this study was undertaken to test whether AET would modulate the UPR in skeletal muscle and whether there is a relationship between UPR markers and tolerance to exercise in a model of CKD in rats.

## Materials and methods

### Animal care

A cohort of thirty‐two male Wistar rats (230–250 g) was obtained from the Experimental Models Development Center for Biology and Medicine of Federal University of Sao Paulo (CEDEME/UNIFESP).The animals were housed under controlled environmental conditions (temperature 23°C; 12‐hr light/dark cycle) and had free access to standard laboratory chow (Nuvilab CR1, Nuvital Nutrients, Brazil) and water. The rats were randomly submitted to a sham surgery or five‐sixths nephrectomy (5/6Nx) under anaesthesia with ketamine (100 mg/kg i.p.) plus xylazine (10 mg/kg i.p.). After ventral laparotomy, removal of the right kidney and ligation of two branches of the left renal artery were performed, and infarction of two‐thirds of the left kidney was achieved. Ten days after the operation, the baseline measurements of body weight (BW) were recorded. The rats were separated into the following four groups and assessed 8 weeks later: control sedentary (CS, *n* = 8), control exercise (CE, *n* = 8), 5/6Nx sedentary (NxS, *n* = 8) and 5/6Nx exercise (NxE *n* = 8). This study was carried out in accordance with National Research Council's Guidelines for the Care and Use of Laboratory Animals and was approved by the Ethics in Research Committee at the Federal University of São Paulo (UNIFESP) (process 0385/2012).

### Renal parameters

After prior adaptation period, rats were housed in metabolic cages and a 24‐hr urine sample was collected at least 48 hrs after the last exercise training session to determine creatinine and protein excretion. Immediately after 24 hrs in cages, the rats were killed by decapitation, and trunk blood was collected in tubes containing anticoagulant, kept on ice and protected from light to obtain of plasma. Creatinine levels were determined on plasma to permit estimation of creatinine clearance as glomerular filtration rate index. For determination of creatinine levels was employed commercial Assay kit (Labtest Diagnóstica SA, Lagoa Santa, MG Brazil) and protein levels were evaluated by Coomassie Blue method 19.

### Exercise training and ability motor

Moderate‐intensity AET was performed on a motor treadmill with 20° of inclination, over 8 weeks, 5 days/week. The intensity of exercise was determined to elicit 60% of maximal speed achieved during a graded treadmill exercise protocol 20, which consisted of treadmill running, with increments of 0.3 km/hr every 3 min., until exhaustion of the animal. Exercise capacity, estimated by total distance run, was evaluated at the onset (10 days after the operation), and after the fourth and the eighth weeks of the protocol. All untrained rats were exposed to treadmill exercise (5 min.) three times a week to become accustomed to exercise protocol and handling.

In addition, to verify whether exercise training would improve motor ability in 5/6Nx rats was performed rotarod test (IITC Life Science, Woodland Hills, CA, USA). The animals were placed on the rod, which was rotating at an initial speed of 1 rpm and the speed gradually increased from 1 to 40 rpm over 5 min. The performance of each animal was considered as the time that the rat stayed on the rod (the best individual performance of three successive trials) 21.

### Structural analysis

For analysis of CSA, the tibialis anterior muscle was chosen as it is composed mainly of glycolytic fibres, which are the most affected in CKD 22, 23. The muscles were removed and frozen in liquid nitrogen. The samples were cut transversally in 6‐μm sections in a cryostat (LEICA CM 1100) and stained with haematoxylin and eosin for examination by light microscopy. Image capture and analysis were performed in a computer‐assisted morphometric system (Leica Quantimet, Cambridge, United Kingdom) with 200× magnification and 20x objective. Images were further analysed on a digitalizing unit connected to a computer using the Quantimet program (Leica Qwim). A minimum of 300 fibres in five different fields were analysed for each animal. All analyses were conducted by a single observer (EM), blinded to the rat's identity.

### Immunoblotting

Bradford assays were used to determine protein concentrations. Tibialis anterior tissue homogenates were subjected to separation by SDS‐PAGE in 8% polyacrylamide gel. After electrophoresis, proteins were electrotransferred to nitrocellulose membrane using the Transblot Semi Dry Transfer Cell (Bio‐Rad Biosciences, Hercules, California, USA). The membranes were then blocked with 0.05% Tween‐20, 10 mM Tris, pH 7.5, 100 mM NaCl (TBST)+5% milk for 1 hr and were rotated overnight at 4°C with the following primary antibodies: phospho‐PERK (Thr980), GRP 78, CHOP, Bcl‐Xl, phospho‐JNK (Thr183/Tyr185), JNK, phospho‐GSK‐3β (Ser9), phospho‐AKT (Ser473), AKT, HSP27 and HSP70 (Cell Signaling Technology Inc., Danvers, Massachusetts, USA); anti‐phospho‐eIF2α (Ser51), ATF6, and phospho‐BAD (Ser99) (Abcam, Cambridge, Massachusetts, USA); anti‐XBP1, Derlin 1, eIF2α and ubiquitinated proteins (Santa Cruz Biotechnology, Santa Cruz, California, USA); and GSK3β and GAPDH (Thermo Fisher Scientific Inc., Waltham, Massachusetts, USA). The immunoblots were washed three times with TBST and incubated for 1 hr with HRP‐conjugated anti‐rabbit secondary antibody (Cell Signaling Technology Inc. Danvers, Massachusetts, USA), before three final TBST washes and incubation with ECL. Quantification of blots was performed with the use of Scion Image software (Informer Technologies Inc., Frederick, Maryland, USA). Protein levels were normalized against GAPDH or Ponceau S staining for polyubiquitin.

### Slot blot

Proteins were quantified as described in immunoblotting analysis. Tibialis anterior tissue homogenate (25 μg protein) was slot‐blotted onto PVDF membrane, and membranes were washed three times with 0.05% Tween‐20, 10 mM Tris, pH 7.5, 100 mM NaCl (T‐TBS) and blocked in T‐TBS+5% milk. Membranes were incubated with an antisoluble oligomer antibody (Biosource International, Camarillo, California, USA) that recognizes misfolded proteins by exposition to hydrophobic sites. Sample loading was normalized by Ponceau staining 17, 24.

### Assay of 26S proteasome activity

Chymotrypsin‐like activity of proteasome was assayed in the total lysate from tibialis anterior using the fluorogenic peptide Suc‐Leu‐Leu‐Val‐Tyr‐7‐amido‐4‐methylcoumarin (Biomol International, Farmingdale, New York, USA). Peptidase activities were measured as previously described 25. The assay was performed in a microtiter plate, in assay buffer containing 25 mM Tris–HCl, 5.0 mM MgCl2, 25 μM ATP, pH 7.5. Kinetic analyses were carried out using 50 μg of protein for 30 min. at 37°C in the presence and absence of 1 μM epoxomicin, with the difference attributed to ATP‐dependent proteasomal activity. Excitation/emission wavelengths were 350/440 nm, respectively.

### Oxidative stress measurement

#### Comet assay

The protocol established for single cell gel (comet) assay for skeletal muscle cells was used, with some modifications 26. Central fragments from skeletal muscle were collected and minced in 0.9% NaCl using a wood spatula. The resulting suspensions were centrifuged at 800 rpm for 5 min. and were added to 120 μl of 0.5% low‐melting‐point agarose at 37°C, layered onto a pre‐coated slide with 1.5% regular agarose and covered with a coverslip. After brief agarose solidification in refrigerator, the coverslip was removed and slides immersed in lysis solution (2.5 M NaCl, 100 mM EDTA, 10 mM Tris–HCl buffer, pH 10,1% sodium sarcosinate with 1% Triton X‐100 and 10% DMSO), for about 1 hr. Prior to electrophoresis, the slides were left in alkaline buffer (pH > 13) for 20 min. and electrophoresed for another 20 min., at 0.7 V/cm, 300 mA. After electrophoresis, the slides were neutralized onto 0.4 M Tris–HCl (pH 7.5), fixed in absolute ethanol and stored until analysis. DNA was stained by adding 100 μl ethidium bromide (50 μg/ml) onto each slide. To minimize extraneous DNA damage from ambient ultraviolet radiation, all steps were performed with reduced illumination.

A total of 25 randomly captured comets per animal were examined blindly by one expert observer at 400× magnification using a fluorescence microscope (Olympus) connected through a black and white camera to an image analysis system (Comet Assay II, Perceptive Instruments, Suffolk, Haverhill, UK) calibrated previously according to manufacturer's instructions. The computerized image analysis system acquires images, computes the integrated intensity profiles for each cell, estimates the comet cell components and then evaluates the range of derived parameters. Undamaged cells have an intact nucleus without a tail, and damaged cells have the appearance of a comet. To measure DNA damage, the tail intensity (% migrated DNA) was considered 26.

#### Lipid peroxidation

The samples were homogenized (1:10 w/v) in cold phosphate‐buffered saline (100 mM, pH 7.4) and centrifuged at 12 000 *g* for 20 min. at 4°C. Proteins were precipitated with trichloroacetic acid (10% w/v), and supernatant was mixed with ferrous oxidation–xylenol orange (FOX) reagent and incubated for 30 min. and absorbance was read at 560 nm 27.

#### Carbonylated protein levels

This analysis was performed as described by Antony *et al*. 28, where the carbonyl group of side chains of proteins reacts with 2,4‐dinitrophenylhydrazine resulting in 2,4‐dinitrophenylhydrazone. After this step, proteins were loaded in gel electrophoresis as described in immunoblotting section. The OxyBlot Protein Oxidation Detection Kit (Millipore, Burlington, Massachusetts, USA) was assessed, following manufacturer's instruction.

#### Adducts of 4‐hydroxynonal

The aldehyde 4‐hydroxy‐2‐nonenal (4‐HNE) is a major end product of peroxidation of membrane ω‐6‐polyunsaturated fatty acids. As 4‐HNE has important electrophilic properties and reacts with many classes of biomolecules resulting in adducts, it can be detected using antibodies that recognize adducts of 4‐HNE. These covalent adducts can be detected using specific anti‐4‐HNE (Calbiochem, HE, Darmstadt, Hessen, Germany). The proteins were quantified and loaded in gel electrophoresis and analysed as described in immunoblotting section.

#### Antioxidant enzymatic essays

The assays for enzymes catalase (CAT) and glutathione peroxidase (GPx) were performed to investigate cell defence antioxidant. The samples were homogenized in a solution containing 440 mM cold sucrose, 50 mM MOPS, 0.01 mM phenylmethylsulfonyl fluoride (PMSF) and 100 mM EDTA (pH 7.2) followed by centrifugation at 750× g for 10 min. at 4°C. The aliquots of the supernatant were collected and used for proteins quantification as described in immunoblotting section. For CAT activity, 50 mM phosphate buffer was utilized and measured in pH 7.0 by monitoring the decrease in absorbance at 240 nm for 30 sec. after the addition of 10 mM hydrogen peroxide. One unit of catalase activity is the amount of enzyme present that decomposes 1 μM H_2_O_2_/min. at 25°C 29. For GPx, 0.1 M phosphate buffer (pH 7.0) was utilized and activity was assayed by following NADPH oxidation at 340 nm; calculations were based on the rate of reaction over the first 4 min. following an addition of hydrogen peroxide in the presence of glutathione reductase, glutathione and tert‐butyl hydroperoxide as substrates and addition of sodium azide 30.

### Quantitative PCR

Total RNA was isolated from tibialis anterior muscles using TRIzol (Invitrogen; Carlsbad, CA, USA). RNA concentration and integrity were assessed. Reverse transcription was performed using Revertaid first‐strand cDNA synthesis kit (Fermentas; Glen Burnie, MD, USA) at 70°C for 10 min., followed by incubation at 42°C for 60 min. and at 95°C for 10 min. SYBR Green/ROX qPCR Master Mix (Applied Biosystems, Waltham, Massachusetts, USA) was used to detect mRNA levels of GRP78 (Fw 5‐ACGTGTCTTGGGCTCAGGGAGAGGAG‐3, Rv 5‐GTTCCTACACCAGATGTGCATGACCCAAC ‐3′), CHOP (Fw 5′‐CCTAGCTTGGCTGAGAGAGG‐3′, Rv 5′‐CTGCTCCTTCTCCTTCATGC‐3′), ATF4 (Fw 5′‐GAGCTTCCTGAACAGCGAAGTG‐3′, Rv 5′‐TGGCCACCTCCAGATAGTCATC‐3′) and XBP1 (Fw 5′‐GGGAATGGAGTAAGGCTGG‐3′, Rv 5′‐TCAGAATCTGAAGAGGCAACA‐3′) using cyclophilin A (Fw 5′‐TGG CAA GCA TGT GGT CTT TGG GAA G‐3′, Rv 5′‐ GGT GAT CTT CTT GCT GGT CTT GCC ATT C‐3′) as housekeeping. All amplification plots were detected by ABY Prism 7500 Sequence Detection System (Applied Biosystems), and gene expression was calculated using ΔΔCt method. Control rat average values were considered as 1.

### Statistical analysis

AET's effects were tested by analysis of variance (anova) one or two way, as appropriate. If statistically significant differences were detected by anova,* post hoc* comparisons between groups were performed using the *Student–Newman–Keuls test* and were considered a significance level of *P* ≤ 0.05.

## Results

### Renal parameters

Renal parameters of rats are presented in Table 1. No significant differences were seen between CS and CE groups. For the 5/6NxS group, higher levels of urinary protein and protein: creatinine ratio and lower values of creatinine clearance were observed compared to control groups, evidence of worsening renal function. In contrast, 5/6NxE demonstrated marked reduction in urinary protein and protein: creatinine ratio levels, indicating improvement in renal function. In addition, renal hypertrophy index was also substantially increased in 5/6NxS animals, demonstrating hypertrophy of the remaining nephrons. Despite higher median values of this index in 5/6NxE, there were no statistically significant differences compared to 5/6NxS.

**Table 1:** Protein urinary, creatinine clearance, protein:creatinine ratio and kidney hypertrophy index in control (CS), exercise control (CE) and 5/6 nephrectomy sedentary (5/6NxS) or exercise 5/6Nx (5/6NxE)

Parameter	CS	CE	5/6NxS	5/6NxE
Protein (mg/24 hrs)	17.6 ± 2.7	18 ± 2.8	92.2 ± 11.0† ^,^ ‡	42.2 ± 6.3† ^,^ ‡ ^,^ §
Creatinine clearance (ml/min.)	1.7 ± 0.3	1.8 ± 0.3	0.9 ± 0.2† ^,^ ‡	1.3 ± 0.2
Protein: creatinine	1.6 ± 0.3	1.4 ± 0.3	3.8 ± 0.4† ^,^ ‡	2.4 ± 0.4† ^,^ ‡ ^,^ §
Hypertrophy index* (g/cm)	32.3(28.9–33.6)	33.3(31.7–35.7)	55.48(44.4–63.4)† ^,^ ‡	49.3(42.8–56.6)† ^,^ ‡

*Median values (interquartile intervals). Kolmogorov–Smirnov normality test and Wilcoxon signed‐rank test.

*n* = 8/per group. ^†^ *P* < 0.05 *versus* CS; ^‡^ *P* < 0.05 *versus* CE; ^§^ *P* < 0.05 *versus* 5/6NxS

### Exercise capacity and body weight

**Figure 1:**
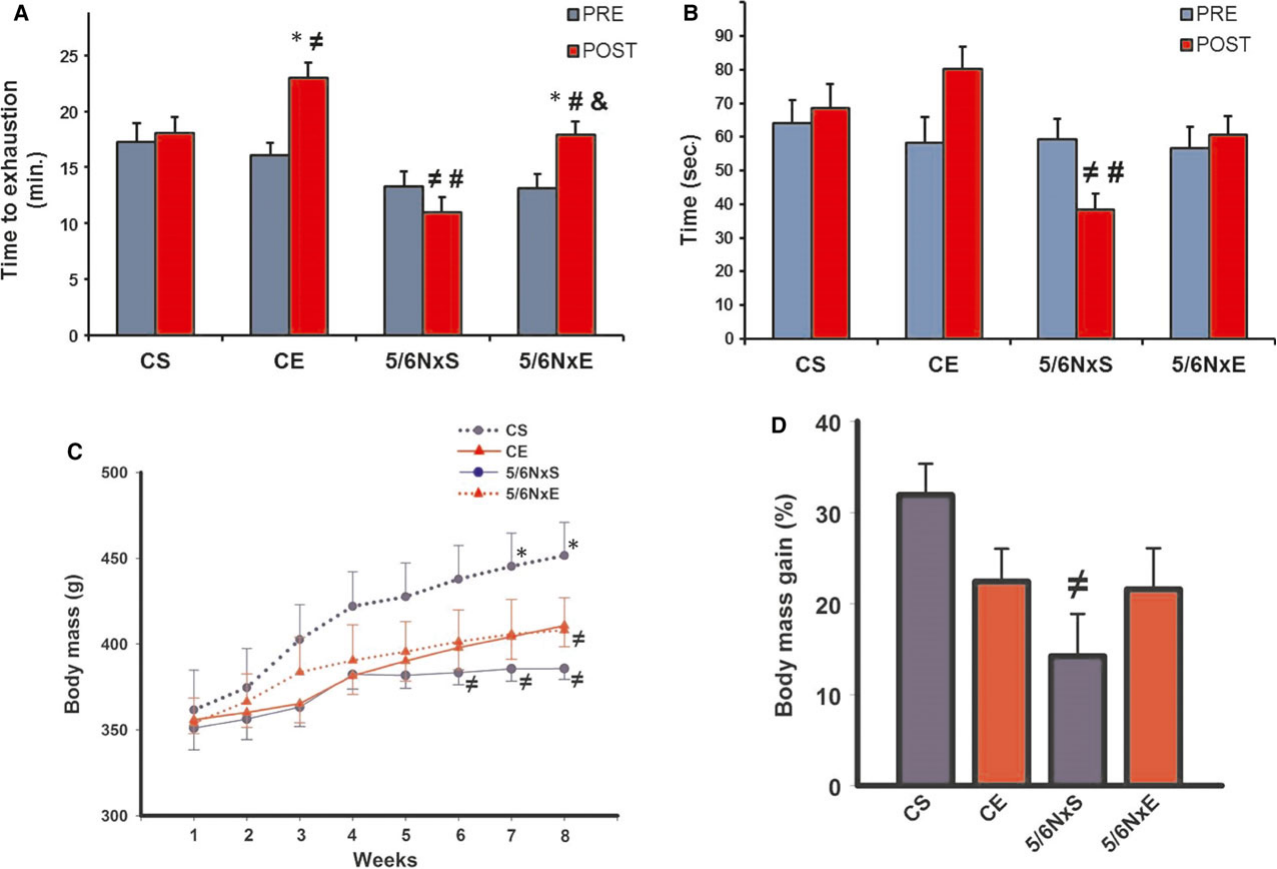
Exercise tolerance (**A**), rotarod test (**B**) and temporal changes in body mass (**C**), body mass gain (**D**) in control (CS), exercise control (CE) and 5/6 nephrectomy sedentary (5/6NxS) or exercise 5/6Nx (5/6NxE).*P < 0.05 *versus* Pre; ^≠^ *P* < 0.05 *versus* CS; ^#^ *P* < 0.05 *versus* CE; ^&^ *P* < 0.05 *versus* 5/6NxS. Data are presented as mean ± S.E. *n* = 8/per group.

Nephrectomized (5/6NxS) rats displayed less exercise tolerance when compared with CS and CE in the postexperimental period, suggesting a rapid deleterious effect of CKD in motor performance (Fig. 1A). In turn, AET improved exercise tolerance in both CE and 5/6NxE groups, suggesting that CKD does not attenuate the improvement induced by AET.

To confirm these results, another test of motor ability, rotarod, was performed (Fig. 1B). It was observed that 5/6NxS animals had lower performance in the postexperimental period in comparison with CS and CE. The AET was able to restore values of 5/6NxE to a similar degree as that seen in the postperiod CS and CE groups.

Body weight was measured for each group throughout the experiment (Fig. 1C and D). The 5/6NxS group had a smaller body weight compared to CS from the sixth week, and 5/6NxE had lower body weights in relation to the CS group at eighth week (Fig. 1C). When observing the per cent gain in body mass, the 5/6Nx group was significantly lower compared to CS, suggesting that the CKD model showed significant impact in this parameter. The CS, CE and 5/6NxE groups showed no significant differences (Fig. 1D).

### CSA and muscle weight

**Figure 2:**
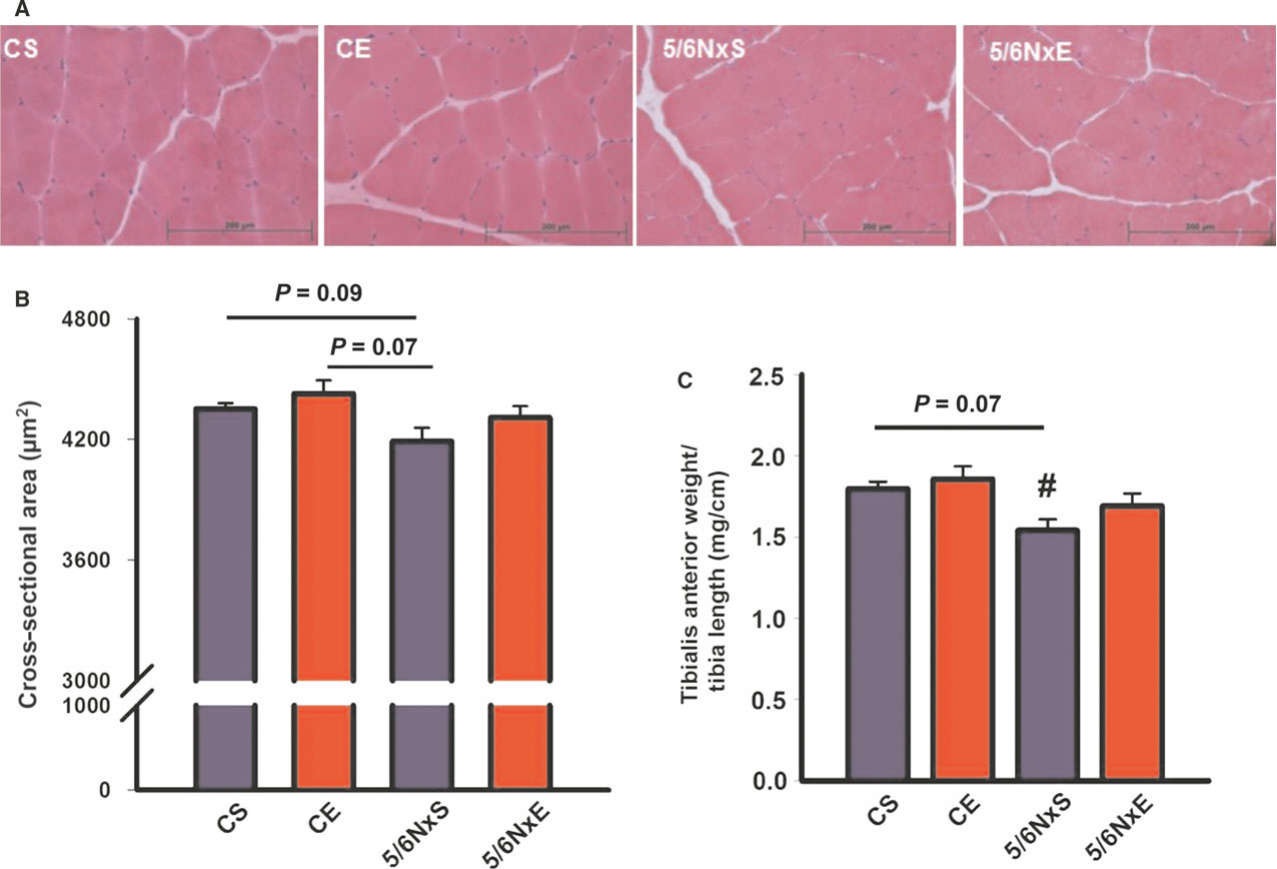
Representative microphotographs of cross‐sectional area (CSA) (**A**) and CSA in tibialis anterior muscle (**B**), tibialis anterior weight/tibial length (**C**) in control (CS), exercise control (CE) and 5/6 nephrectomy sedentary (5/6NxS) or exercise 5/6Nx (5/6NxE). Data are presented as mean ± S.E. *n* = 6/per group.

The quantitative histological analysis (Fig. 2A and B) presented a trend towards decreased CSA in 5/6NxS (*P* = 0.09 *versus* CS and *P* = 0.07 *versus* CE), also evidenced through the lower ratio muscle weight/tibia length (5/6NxS *versus* CS, *P* = 0.07), and significant differences were detected in ratio muscle weight/tibia length in 5/6NxS *versus* CE (Fig. 2C). These data suggest a trend towards muscle atrophy in 5/6Nx, and this tendency was absent when nephrectomized animals trained.

## 26S proteasome activity and accumulated misfolded proteins

**Figure 3:**
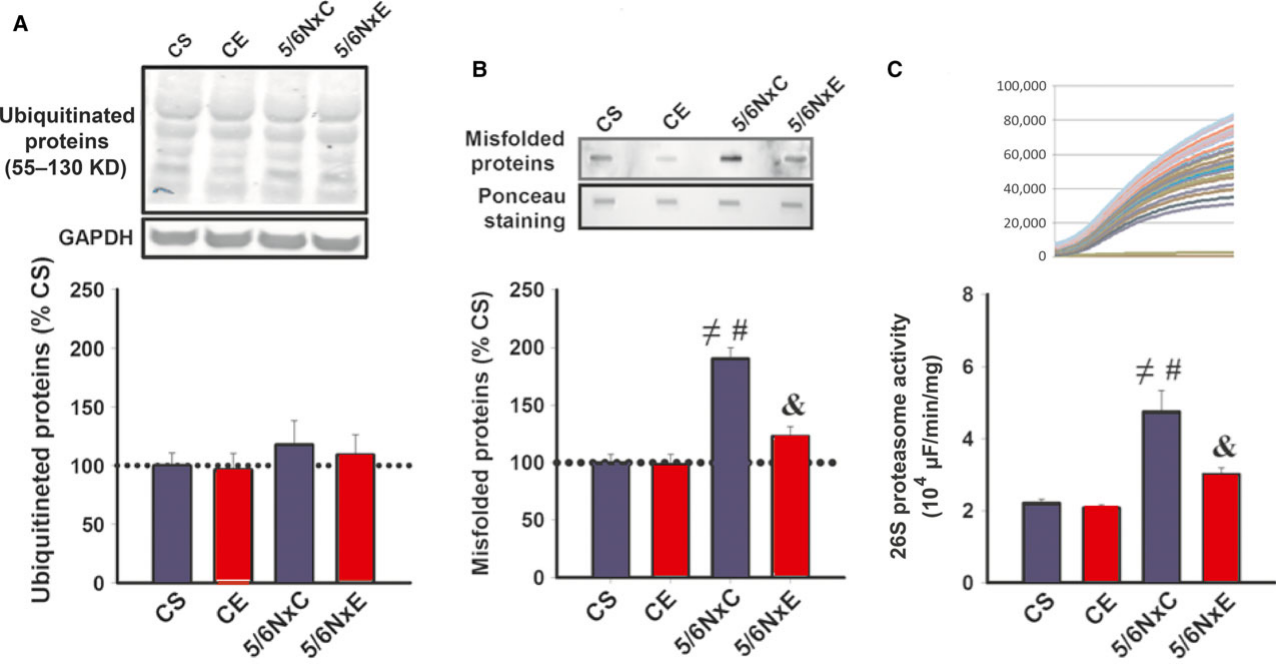
Levels of ubiquitinated (**A**) and misfolded proteins (**B**), and 26S proteasome activity (**C**) in control (CS), exercise control (CE) and 5/6 nephrectomy sedentary (5/6NxS) or exercise 5/6Nx (5/6NxE). ^≠^ *P* < 0.05 *versus* CS; ^#^ *P* < 0.05 *versus* CE; ^&^ *P* < 0.05 *versus* 5/6NxS. Data are presented as mean ± S.E. *n* = 8/per group.

The levels of misfolded and ubiquitinated proteins, as well as changes in proteasomal activity, were assessed (Fig. 3). Ubiquitinated protein levels did not differ between the groups (Fig. 3A). However, 5/6NxS showed a two‐fold increase in misfolded protein levels in comparison with CS group, which was completely restored in 5/6NxE (Fig. 3B).

As the UPS is a major proteolytic pathway responsible for the disposal of misfolded proteins, the chymotrypsin‐like proteasome activity was assessed for each group (Fig. 3C). The 5/6NxS rats showed a significant increase in proteasome activity when compared with the CS and CE groups. Of interest, proteasome activity was effectively reduced to CS levels in 5/6NxE (Fig. 3C).

### Exercise training and cell defence mechanisms in CKD

**Figure 4:**
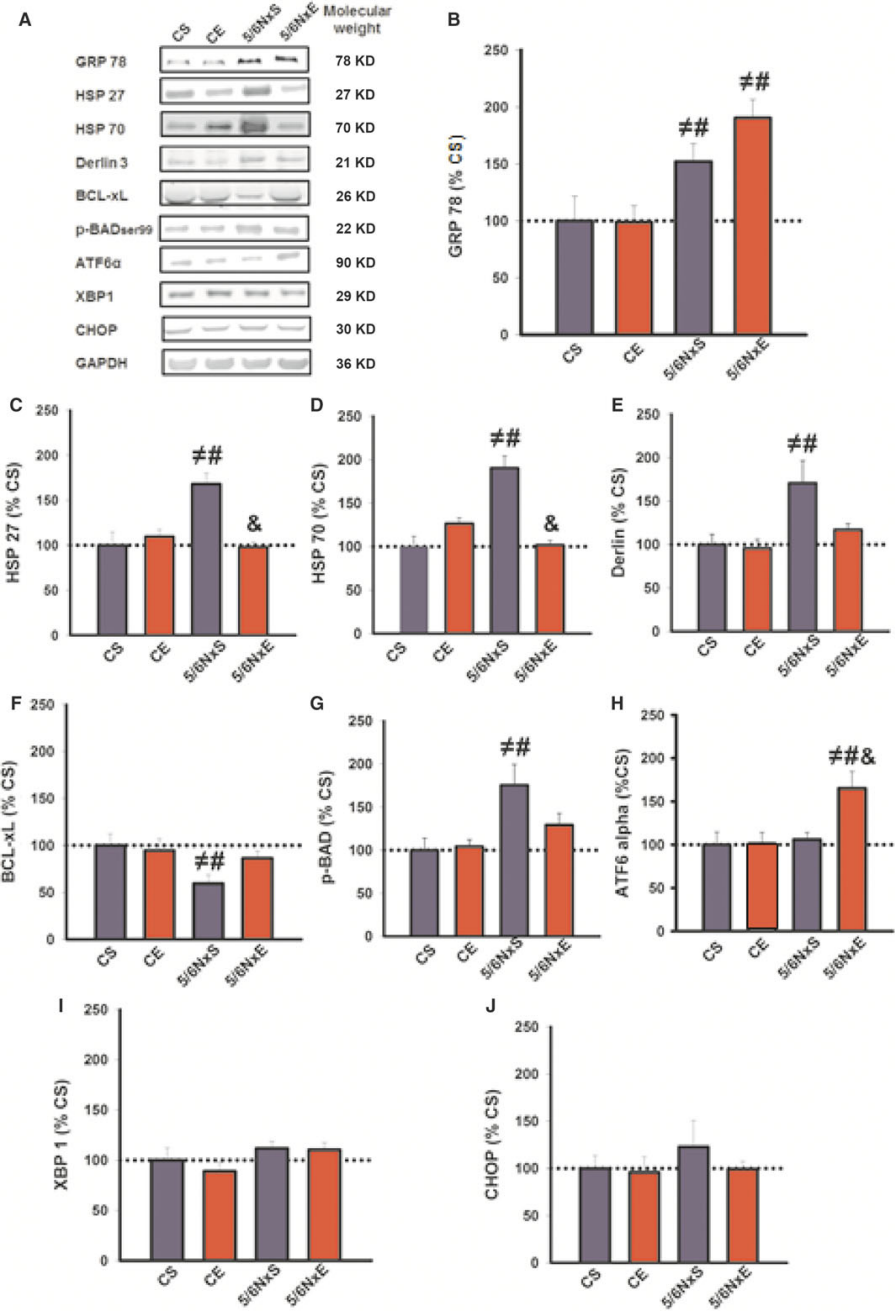
Representative immunoblots (**A**) and quantifications of GRP78 (**B**), HSP27 (**C**), HSP70 (**D**), Derlin 1 (**E**), BCL‐xL (**F**), p‐BAD (**G**), ATF6α (**H**), XBP1 (**I**) and CHOP (**J**) in control (CS), exercise control (CE) and 5/6 nephrectomy sedentary (5/6NxS) or exercise 5/6Nx (5/6NxE). ^≠^ *P* < 0.05 *versus* CS; ^#^ *P* < 0.05 *versus* CE; ^&^ *P* < 0.05 *versus* 5/6NxS. Data are presented as mean ± S.E. *n* = 8/per group.

To investigate whether exercise training improves cellular defence mechanisms in experimental CKD, the protein and activity levels related to UPR, anti‐apoptotic proteins, oxidative damage and antioxidants were evaluated through immunoblotting and enzymatic assays, respectively (Fig. 4). Examining the chaperone system, protein levels of GRP78 (Fig. 4B), HSP27 and HSP70 (Fig. 4C and D), and derlin 1 (Fig. 4E) were higher in 5/6NxS when compared to CS and CE groups. AET restored HSP27, HSP70 and derlin 1, but not GRP78 levels to that of the CS and CE groups. In 5/6NxS animals, a drop in anti‐apoptotic protein Bcl‐xL and an increase in p‐BAD were observed, compared to CS and CE. AET restored both proteins to CS and CE levels (Fig. 4F and G). Interestingly, ATF6α protein levels were higher in 5/6NxE animals than in the other groups (Fig. 4H), suggesting a post‐training adaptation. In contrast, XBP1 and CHOP protein levels had no differences between groups (Fig. 4I and J).

**Figure 5:**
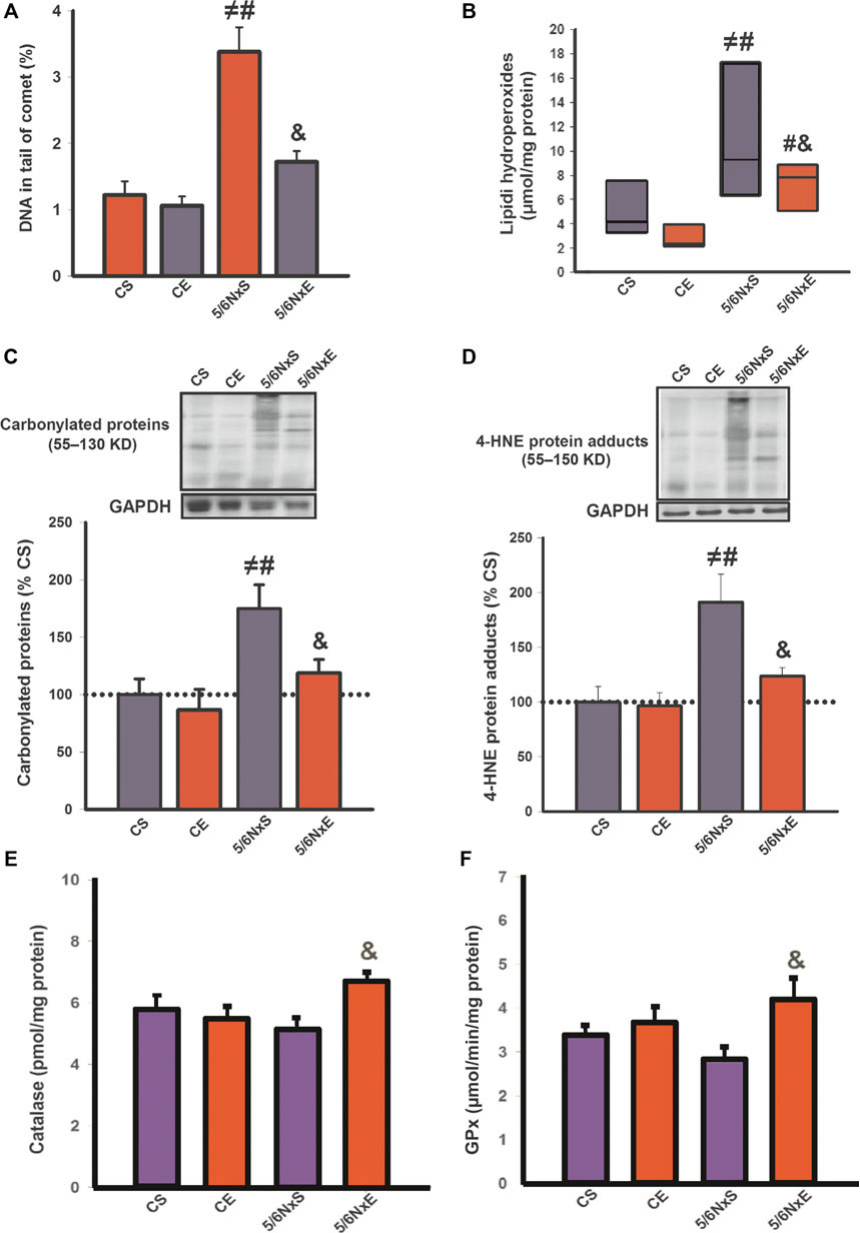
Damage to DNA (**A**), lipid hydroperoxidation (**B**), carbonylated protein (**C**), 4‐HNE protein adducts (**D**), catalase activity (**E**) and GPx activity (**F**) in control (CS), exercise control (CE) and 5/6 nephrectomy sedentary (5/6NxS) or exercise 5/6Nx (5/6NxE). ^≠^ *P* < 0.05 *versus* CS; ^#^ *P* < 0.05 *versus* CE; ^&^ *P* < 0.05 *versus* 5/6NxS. Data are presented as mean ± S.E. *n* = 8/per group.

It is well known that oxidative stress is increased in CKD and that AET is capable of increasing antioxidant defence systems to mitigate oxidative damage. We therefore wanted to see whether AET could mitigate the oxidative damage in CKD. DNA damage, evaluated by the comet assay, may reflect both oxidative and apoptotic processes. In the tibialis anterior muscle, DNA damage, as measured by the length of the comet's tail, was higher in the 5/6NxS group compared to the CS and CE groups (Fig. 5A). In addition, oxidative stress, measured by the levels of lipid hydroperoxides (Fig. 5B), carbonylated proteins (Fig. 5C) and 4‐HNE protein adducts (Fig. 5D), was increased in 5/6Nx in comparison with CS and CE groups. Interestingly, AET decreases these levels in 5/6NxE. When examining the activity of the antioxidant enzymes catalase and GPx, activity was increased in the 5/6NxE group compared to 5/6NxS (Fig. 5F and G). These data show that AET can effectively attenuate the oxidative damage in skeletal muscle associated with CKD, potentially through elevating the activity of antioxidant enzymes. Together, these data reinforce that exercise training is able to improve multiples cellular defence mechanisms in CKD.

### Exercise training and protein synthesis/pro‐survival signalling pathways in CKD

**Figure 6:**
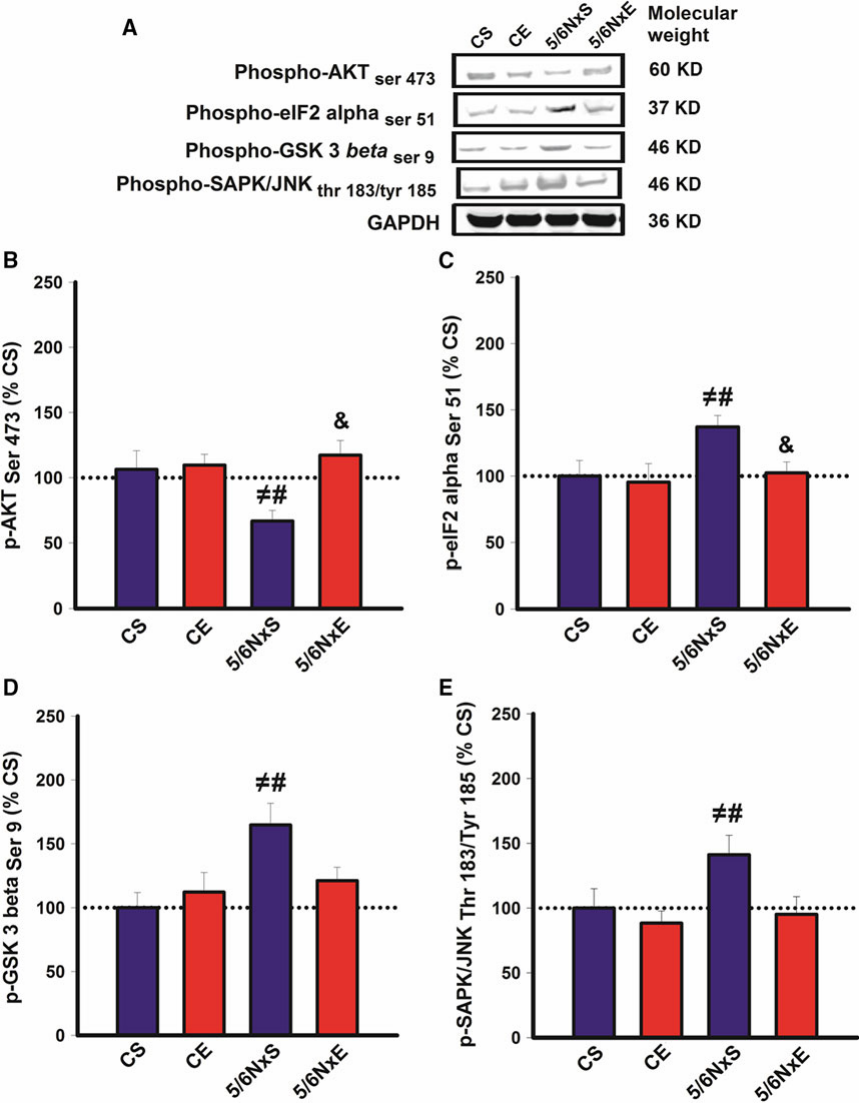
Representative immunoblots (**A**) and protein levels of p‐AKT (**B**), p‐GSK3β (**C**), p‐eIF2‐α (**D**) and p‐JNK (**E**) in control (CS), exercise control (CE) and 5/6 nephrectomy sedentary (5/6NxS) or exercise 5/6Nx (5/6NxE). ^≠^ *P* < 0.05 *versus* CS; ^#^ *P* < 0.05 *versus* CE; ^&^ *P* < 0.05 *versus* 5/6NxS. Data are presented as mean ± S.E. *n* = 8/per group.

To investigate whether AET affects protein synthesis or pro‐survival signalling, we used Western blot analysis to look at the levels of proteins associated with both pathways (Fig. 6). Total levels of AKT, eIF2‐α, GSK3β and JNK did not change between groups (data not shown). A decrease in p‐AKT (Fig. 6B) and increases in p‐eIF2‐α, p‐GSK3β and p‐JNK were observed in the 5/6NxS group compared to CS and CE (Fig. 6C–E). These data are consistent with reduced global protein synthesis and pro‐survive pathways in CKD. Interesting to note, AET restores the levels of these proteins in nephrectomized animals back to the level of control animals (Fig. 6A–E), suggesting exercise training plays an important role in re‐establishing theses pathways in CKD.

### Exercise training and genes related to UPR in CKD

**Figure 7:**
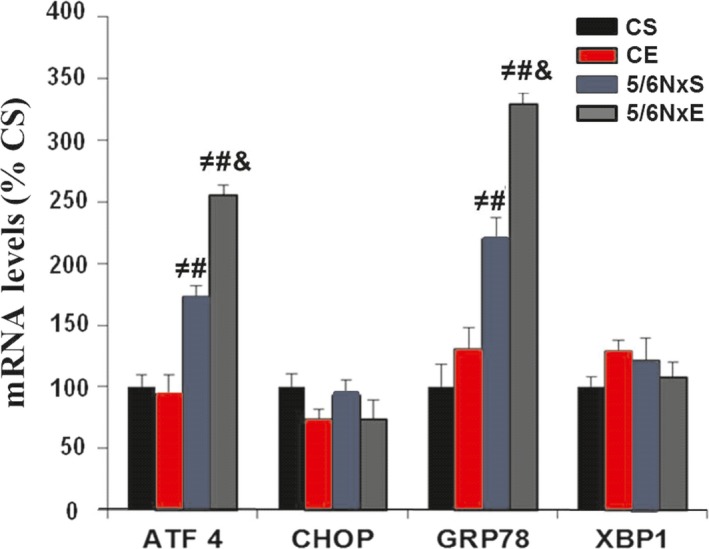
mRNA levels in control (CS), exercise control (CE) and 5/6 nephrectomy sedentary (5/6NxS) or exercise 5/6Nx (5/6NxE). ^≠^ *P* < 0.05 *versus* CS; ^#^ *P* < 0.05 *versus* CE; ^&^ *P* < 0.05 *versus* 5/6NxS. Data are presented as mean ± S.E. *n* = 8/per group.

qPCR analysis was used to examine the expression of UPR‐related genes (Fig. 7). No differences were seen in XBP1 and CHOP expression between groups. In 5/6Nx, expression of ATF4 and GRP78 genes were elevated compared to CS and CE, with AET resulting in an even greater increase in these genes, suggesting amplification in UPR in 5/6NxE.

## Discussion

In the present study, the effects of a moderate‐intensity protocol of AET on skeletal muscle UPR in a rat model of CKD were assessed. Despite extensive literature highlighting exercise tolerance and the importance of preserving the muscle tissue integrity, this is the first study that provides approaches to the UPR in skeletal muscle utilizing CKD models, especially as part of exercise training‐induced adaptation.

5/6Nx model displayed similarities with clinical conditions observed in humans with CKD, showing progressive decline in renal function and reductions in body weight gain. In addition, the present research confirmed that these clinical conditions associated with CKD led to disruption of normal skeletal muscle homeostasis, leading to accumulation of misfolded proteins, increased apoptotic markers and oxidative damage. On the other hand, the AET protocol used in the present study was able to slow the progression of renal dysfunction, reducing proteinuria and increasing creatinine clearance, similar to reports from previous studies using the 5/6Nx model, using different protocols of AET 31, 32. In skeletal muscle, AET prevented the drop in exercise capacity followed by UPR activation, preventing misfolded proteins accumulation and attenuating pro‐apoptotic markers and oxidative damage, with a tendency to change in fibre size.

If the initial purpose of the UPR is to adapt the ER to environmental changes and restore its function, it can be speculated that this response in skeletal muscle of 5/6Nx is not sufficient as an accumulation of misfolded protein occurred. In accordance with UPR activation, a deficit in the signalling involved in protein synthesis, which is intended to inhibit the synthesis of new polypeptide chains, as well as a hyperactivation of the UPS, for increase cell capacity to degrade proteins 11, was observed. The misfolded proteins in the ER are relocated to the cytosol to be degraded by the proteasome. This process is mediated by derlin 1 24, which was elevated in 5/6NxS, probably reflecting an excess of substrates for the UPS which originated in ER. In addition, other researchers have also reported down‐regulation in the AKT pathway and hyperactivation of the UPS in 5/6Nx 33, 34. AET prevented misfolded protein accumulation, re‐establishing UPS activation and derlin 1 protein levels as observed in 5/6NxE, reflecting a lower proteasomal load induced by unfolded/misfolded substrates.

Another remarkable feature in CKD is apoptosis of skeletal muscle 35. In the present study, although increased protein and gene expression of CHOP were not found, apoptotic signalling was altered, as noted by increased p‐BAD and decreased anti‐apoptotic Bcl‐xL protein levels in 5/6Nx, suggesting that apoptosis is CHOP activation independent. In fact, several pathways may be involved in apoptosis, such as that mediated by JNKs activation. Corroborating this, increased levels of p‐JNK, an indicator of protein activation, were observed in the present study. JNK can phosphorylate residues in BAD and Bcl‐xL, as well as lead to transcription of several transcription factors that contribute to apoptosis 36. Apoptotic signalling precedes the degradation of proteins during muscular atrophy in CKD animals and patients 35, which could explain the pro‐apoptotic signalling and hyperactivation of the UPS 33, 34 without evidence of muscle atrophy in the locomotor muscles in the 5/6Nx model 37, 38. Our findings corroborate these results, as 5/6NxS show a tendency to muscle atrophy, probably reflecting a moderate stage of disease. On the other hand, it can be argued that, in this stage, interventions should be prioritized as current therapeutic interventions have limited clinical impact on CKD when in advantaged stages 39.

Here, AET was able to attenuate apoptosis and reactive AKT pathway signalling in accordance with previous findings in 5/6Nx 40 and CKD patients 41, confirmed by reduced DNA fragmentation in the comet assay. The ‘pro‐survival’ role of AKT activation appears to be, at least in part, due to its ‘anti‐apoptotic’ role. Activated AKT can phosphorylate BAD and lead to phosphorylation of GSK‐3β (glycogen synthase kinase‐3β). BAD and GSK‐3β exert no apoptotic function in the phosphorylated state, preventing apoptosis 42.

In parallel, we showed an increase in oxidative stress in 5/6Nx, a remarkable feature in CKD 5, by several parameters of structural oxidative damage, such as lipid peroxidation, accumulated 4HNE adducts and carbonylated proteins levels, DNA disruption and reduced activity of antioxidants GPx and catalase. These data suggest that skeletal muscle of 5/6NxS is exposed to chronic oxidative stress and are not be able to adapt to this stress induced by reactive species generation. In addition, HSPs 27 and 70 protein levels, which are synthesized in response to oxidative stress, were elevated in 5/6Nx. HSPs have been considered a complementary protection against oxidative stress as an increase in HSP content has been observed under decreased activity of antioxidant enzymes 43, 44. HSP induction also occurs to reorganize the folding of damaged proteins. However, it can be argued that an increase in muscle HSP content is insufficient in 5/6Nx because an accumulation of misfolded proteins persists in 5/6Nx. In agreement with other studies 43, 44, 45, our results indicated an increase in antioxidant capacity post‐training, measured by catalase GPx activity, and a reduction in HSP protein levels. In fact, higher activity of pre‐existing antioxidant enzymes induced by AET requires less synthesis of HSPs for efficient inhibition of reactive species attack 43, 44, 46. The present study in skeletal muscle corroborates the findings demonstrated previously, when AET restored HSPs and misfolded protein levels in cardiac myocytes from an animal model of heart failure 17 and suggests that in some diseases, sustained but insufficient activation of HSPs is reversed by AET when there is improvement in antioxidant defence systems. In our study, a decrease in HSP content post‐training differed from what was observed in reticulum resident GRP78, which increased after AET, demonstrating distinct modulation of cellular chaperones.

Despite the initial protection that stress responses can confer, they are a potential cause of muscle deterioration if the response is excessive or sustained. In fact, excessive induction of stress responses, including JNK signalling, UPS activity and oxidative stress, is known to be deleterious and could lead to the loss of muscle integrity and trigger apoptosis. AET has been shown to be an emerging therapy in CKD, as it improved exercise tolerance and skeletal muscle function 12, 13. In skeletal muscle, AET induced UPR activation as an adaptation to ER stress promoted by acute exercise and contributed to enhanced exercise capacity 9. Thus, it can be argued that the AET induced UPR activation in diseases as CKD, which already have a baseline elevation in markers related to UPR (GRP78 and ATF4 mRNA levels and GRP78 protein levels), do not attenuate but amplify the UPR in skeletal muscle 7. These data differ from those found in control animals, which showed an attenuated or abolished stress response to AET, as they start from lower or normal ‘baseline’. Our findings suggest an ATF6 branch activation in 5/6NxE, which is in line with previous findings 7, 9 that showed that an interaction between transcriptional coactivator PGC‐1α (*peroxisome proliferators‐activator receptor gamma coactivator‐1 alpha*) and ATF6 is one of the mechanisms through which AET induced adaptations in UPR 9.

In summary, our data extend the understanding about skeletal muscle disorders in CKD and suggest that the accumulated misfolded proteins from the ER contribute to the total misfolded proteins in white fibre muscles of rats subjected to 5/6Nx, inducing UPR activation. The moderate‐intensity AET amplified the UPR response, prevented accumulation of misfolded proteins, reduced oxidative damage, restored basal HSP protein levels and increased exercise tolerance. Therefore, our findings support moderate AET for maintaining protein folding and preventing proteotoxicity, inducing protective adaptations against cellular stress induced by chronic uraemia and can play a pivotal role in increasing exercise tolerance.

## Conflict of interests

The authors declare no conflict of interests.
